# The prevalence and treatment rate trends of osteoporosis in postmenopausal women

**DOI:** 10.1371/journal.pone.0290289

**Published:** 2023-09-26

**Authors:** Xiaojuan Zhang, Zheng Wang, Di Zhang, Dandan Ye, Yaru Zhou, Jing Qin, Yingze Zhang

**Affiliations:** 1 The School of Medicine, Nankai University, Tianjin, China; 2 Department of Endocrinology, The Third Hospital of Hebei Medical University, Shijiazhuang, Hebei, China; 3 Department of Spinal Surgery, The Third Hospital of Hebei Medical University, Shijiazhuang, Hebei, China; 4 NHC Key Laboratory of Intelligent Orthopeadic Equipment, The Third Hospital of Hebei Medical University, Shijiazhuang, Hebei, China; 5 Department of Orthopaedics, The Third Hospital of Hebei Medical University, Shijiazhuang, Hebei, China; 6 Faculty of Medicine and Health, Chinese Academy of Engineering, Beijing, China; Universitair Kinderziekenhuis Koningin Fabiola: Hopital Universitaire des Enfants Reine Fabiola, BELGIUM

## Abstract

**Background:**

To evaluate the prevalence and treatment of postmenopausal women with osteoporosis in recent years, analyze differences between the prevalence diagnosed by physicians and the prevalence detected by bone mineral density (BMD), and observe the trends of prevalence and treatment rate of osteoporosis in postmenopausal women over time are of great value for the management of osteoporosis.

**Methods:**

This cross-sectional study collected the data of 4012 postmenopausal women from the National Health and Nutrition Examination Survey (NHANES) from 2005 to 2010, 2013 to 2014 and 2017 to 2018. The prevalence of osteoporosis and osteopenia as well as the treatment rate of osteoporosis were analyzed using Mann-Kendall trend test. Subgroup analysis was conducted in different age, race, body mass index (BMI), diabetes, hypertension, or glucocorticoid use groups.

**Results:**

The overall prevalence of physician diagnosed of osteoporosis was 17.4% and was fluctuated in a small range and remained relatively stable within a certain range (Mann-Kendall trend test: Z = 2.20, *P* = 0.027) during 2005–2018. The prevalence of osteoporosis in postmenopausal women determined by bone mineral density (BMD) examination reached 9.2% during the five cycles. From 2005 to 2018, the prevalence of physician diagnosed osteoporosis fluctuated in a small range. For osteopenia measured by BMD, the prevalence was 59.6% and a gradual increasing trend was found between 2005 and 2018 (Mann-Kendall trend test: Z = 2.20, *P* = 0.027). Among patients with physician diagnosed osteoporosis, the treatment rate reached 70.49%. The treatment rate of physician diagnosed osteoporosis was decreased from 2005 to 2008, and further decreased from 2009 to 2018 (Mann-Kendall trend test: Z = -2.20, *P* = 0.027). The actual treatment rate of osteoporosis patients was 55.53%. During 2005–2018, the actual treatment rate of osteoporosis showed a continuous decline (Mann-Kendall trend test: Z = -2.20, *P* = 0.027).

**Conclusion:**

Osteoporosis management might be insufficient and more efforts are needed to improve the diagnosis and treatment rates of osteoporosis in postmenopausal women.

## Introduction

Osteoporosis is a kind of skeletal disorder characterized by decreased bone quality and bone mineral density [1]. Osteoporosis is associated with elevated risk of fracture, which has become a growing major public health problem, with an impact on quality and quantity of life that crosses medical, social, and economic lines [2]. There was evidence revealing that osteoporosis influenced more than 200 million people all over the world [3]. The disease is more common in women than in men and especially prevalent among postmenopausal women who experience a decline in levels of endogenous estrogens [4]. A previous study indicated that more than a half of postmenopausal White women would suffer an osteoporotic-associated fracture, and osteoporotic-related fractures have brought a severe burden on women and healthcare services [5]. Clinical or subclinical vertebral fractures are the most common type of osteoporotic fractures, which were reported to be associated with a 5-fold increased risk for additional vertebral fractures and a 2- to 3-fold increased risk for fractures at other sites [6]. To deep explore the prevalence, treatment rate and trend of osteoporosis in postmenopausal women is important.

Recently, although the United States Preventive Services Task Force (USPSTF) recommends routine osteoporosis screening and bone mineral density (BMD) testing for women aged≥65 years [7]. Whether routine osteoporosis screening for postmenopausal women < 65 years is needed remains controversial [7]. Another cross-sectional study depicted that the prevalence of physician-diagnosed osteoporosis was on the rise in both men and women >50 years, while the estimated treatment rate of physician-diagnosed osteoporosis patients has not changed significantly [8]. Additionally, the management of osteoporosis and the fracture prevention strategies are often poorly implemented by clinicians in primary care [9]. Despite the availability of effective anti-fracture interventions and the potentially fatal consequences of fractures, osteoporosis remains a disease that is frequently underdiagnosed and undertreated [10]. Currently, studies evaluating the prevalence and treatment rate of osteoporosis in postmenopausal women are required for increasing the awareness of the diagnosis and treatments of osteoporosis in postmenopausal women.

In our study, we planned to explore the trends of prevalence and treatment rate of osteoporosis and osteopenia in postmenopausal women over time based on the data from the National Health and Nutrition Examination Survey (NHANES). The prevalence and treatment of osteoporosis and osteopenia in postmenopausal women during recent years, the differences between the prevalence of osteoporosis and osteopenia diagnosed by physicians and the prevalence detected by BMD, and the trend of prevalence and treatment rate of osteoporosis in postmenopausal women with different ages, races and other characteristics are valuable for the further targeting management.

## Methods

### Study design and population

This cross-sectional study collected the data of 5309 postmenopausal women in the NHANES from 2005 to 2010, 2013 to 2014 and 2017 to 2018. NHANES is a program of continuous 2-year-cycle cross-sectional surveys conducted by the Centers for Disease Control and Prevention (CDC). NHANES involved in about 5,000 persons each year from various counties across the U.S., which are divided into a total of 30 primary sampling units (PSUs), of which 15 are visited annually. The NHANES combines interviews, physical examinations, and laboratory evaluations to obtain a large amount of quantitative and qualitative data [11]. All participants provided a written informed consents before participation. Household questionnaires, telephone interviews, and examinations conducted by healthcare professionals and trained personnel were utilized to collect data. In the NHANES database, dual-energy X-ray absorptiometry (DXA) examinations of femoral neck and total femur were performed in 2005–2006, 2007–2008, 2009–2010, 2013–2014 and 2017–2018, and these five cycles were included in the present study. Participants without data on the assessment of BMD at the femur neck and total femur, without data on physician diagnosis of osteoporosis, and without data on marriage, smoking, previous fracture, parental fracture, height, weight, waist circumference or total energy were excluded. Finally, 4012 subjects were included. The methods were reported following STROBE-checklist-v4-combined-PlosMedicine.

### Variables and definitions

Age (years), race (non-Hispanic White, non-Hispanic Black or other), education (less than 9th Grade, 9-11th Grade, high school Grad/General Equivalent Diploma (GED)or Equivalent, some college or AA degree, or college graduate or above), marriage (married, widowed, divorced, separated, never married or living with partner), poverty-to-income ratio (PIR), drinking (yes or no), smoking (yes or no), physical activity (MET· min/week), hypertension (yes or no), diabetes (yes or no), previous fracture (yes or no), parental fracture (yes or no), glucocorticoid use (yes or no), anti-osteoporosis therapy (yes or no), body mass index (BMI, <25 kg/m^2^ or ≥25 kg/m^2^), waist circumference (cm), cotinine (ng/mL), cadmium (ug/L), lead (ug/dL), iron (umol/L), mercury (umol/L), 25-hydroxyvitamin D2 [25(OH)D, nmol/L], total energy (kcal), Day 1 calcium intake (mg), calcium intake in dietary supplement (yes or unknown), total calcium intake (mg), Day 1 vitamin D intake (yes, no or unknown), and vitamin D intake in dietary supplement (yes or unknown) were variables analyzed in this study.

Diabetes was defined as those with fasting blood glucose ≥7.0 mmol/L, glycosylated hemoglobin (HbAlc) ≥6.5%, physician diagnosis or those receiving hypoglycemic therapy. Glucocorticoid use was identified according to OSQ (Ever taken prednisone or cortisone daily?) or whether they had used hormonal medications. Smoke was defined based on SMQ_D, and those have smoked at least 100 cigarettes in the lifetime were considered to be a smoker, otherwise a non-smoker. Physical activity was converted into energy expenditure, which was calculated based on PAQ from the database. Energy expenditure [Metabolic Equivalent (MET)·min] = recommended MET × Exercise time of corresponding activity (min).

### Outcome variables

#### The prevalence of physician diagnosed osteoporosis during the 5 cycles

People who answered “yes” to the question “Have you ever been diagnosed with osteoporosis by a physician?” were classified as physician-diagnosed cases of osteoporosis. The number of physician diagnosed cases of osteoporosis during the 5 cycles was divided by the total number of postmenopausal women in the 5 cycles analyzed in our study to calculate the weighted overall prevalence of physician diagnosed osteoporosis. The number of physician diagnosed osteoporosis patients in 2005–2006, 2007–2008, 2009–2010, 2013–2014 or 2017–2018 was divided by the total number of postmenopausal women in each year to calculate the annual weighted prevalence of physician diagnosed osteoporosis.

### The prevalence of osteoporosis diagnosed by BMD measurement

The number of osteoporosis patients diagnosed by BMD measurement during the 5 cycles was divided by the total number of postmenopausal women in the 5 cycles to calculate the measured weighted prevalence of osteoporosis diagnosed by BMD measurement. The numbers of osteoporosis patients diagnosed by BMD measurement in 2005–2006, 2007–2008, 2009–2010, 2013–2014 or 2017–2018 was divided by the total number of postmenopausal women in each year to calculate the weighted prevalence of osteoporosis diagnosed by BMD measurement.

### The prevalence of physician diagnosed osteopenia during the 5 cycles

Women who answered “yes” to the question “Have you ever been diagnosed with osteopenia by a physician?” were classified as physician-diagnosed cases of osteopenia. The number of physician diagnosed cases of osteopenia during the 5 cycles was divided by the total number of postmenopausal women in the 5 cycles analyzed to calculate the weighted overall prevalence of physician diagnosed osteopenia. The number of physician diagnosed osteopenia patients in 2005–2006, 2007–2008, 2009–2010, 2013–2014 or 2017–2018 was divided by the total number of postmenopausal women in each year to calculate the annual weighted prevalence of physician diagnosed osteopenia.

### The prevalence of osteopenia diagnosed by BMD measurement

The number of osteopenia patients diagnosed by BMD measurement during the 5 cycles was divided by the total number of postmenopausal women in the 5 cycles to calculate the measured weighted prevalence of osteopenia diagnosed by BMD measurement. The numbers of osteopenia patients diagnosed by BMD measurement in 2005–2006, 2007–2008, 2009–2010, 2013–2014 or 2017–2018 was divided by the total number of postmenopausal women in each year to calculate the weighted prevalence of osteopenia diagnosed by BMD measurement in each cycle.

### The treatment rate of physician diagnosed osteoporosis

The number of osteoporosis patients received treatments during the 5 cycles was divided by the total number of physician diagnosed osteoporosis postmenopausal women in the 5 cycles to calculate the weighted overall treatment rate of physician diagnosed osteoporosis. The treatment of physician diagnosed osteoporosis was defined based on the answer of “yes” to the question “Have you ever been treated for osteoporosis by a physician?”, and women who received metabolic agents, bone resorption inhibitors, bisphosphonates, and miscellaneous bone resorption inhibitors. The number of osteoporosis patients received treatments in 2005–2006, 2007–2008, 2009–2010, 2013–2014 or 2017–2018 was divided by the total number of physician diagnosed osteoporosis postmenopausal women in each year to calculate the annual weighted treatment rate of physician diagnosed osteoporosis in each year.

### The actual treatment rate of osteoporosis

The number of osteoporosis patients received treatments during the 5 cycles was divided by the total number of postmenopausal women with physician diagnosed osteoporosis and osteoporosis diagnosed by BMD measurement in the 5 cycles to calculate the actual treatment rate of osteoporosis. Women who answered “yes” to the question “Have you ever been treated for osteoporosis by a physician?”, and those who received metabolic agents, bone resorption inhibitors, bisphosphonates, and miscellaneous bone resorption inhibitors were defined to received treatments for osteoporosis. The numbers of osteoporosis patients received treatments in 2005–2006, 2007–2008, 2009–2010, 2013–2014 or 2017–2018 was divided by the total number of postmenopausal women with physician diagnosed osteoporosis and osteoporosis diagnosed by BMD measurement in each year to calculate the weighted actual treatment rate of osteoporosis.

The BMD of the total femur and femur neck was extracted by searching DXA of total femur BMD (DXXOFBMD) or DAX of femur neck BMD DXXNKBMD variable in DXA femur bone (DXXFEM) data file (https://wwwn.cdc.gov/Nchs/Nhanes/2005-2006/DXXFEM_D.htm). All participants underwent BMD testing by DXA examinations. BMD was measured via DXA and BMD at the femur neck and total femur was used to calculate the T-score [respondent’s BMD-reference group mean BMD)/reference group standard deviation (SD)]. The reference group for the femoral neck consisted of non-Hispanic White women aged 20–29 years from NHANES III [12]. Osteoporosis was defined as femur neck or total femur BMD T-score ≤ −2.5. Osteopenia was defined as femur neck or total femur BMD T-score ≤ −1. In this study, BMD T-score ≤ −2.5 at any of the femur neck or the total femur was considered osteoporosis and T-score ≤ −1 at any of the femur neck or the total femur was considered osteopenia.

Physician diagnosed osteoporosis was determined by the answer of “yes” to the question “Has a doctor ever told you that you had osteoporosis, sometimes called thin or brittle bones?” in the osteoporosis section questionnaire 060 (OS1060) and the treatment of osteoporosis was determined by the answer of “yes” to question “Were you treated for osteoporosis?” in the osteoporosis section questionnaire 070 (O SQ070).

### Statistical analysis

The normality of quantitative data was evaluated by Kolmogorov-Smirnov, and normally distributed measurement data were described as Mean ± standard deviation (SD). Independent sample t test was used for comparison between the two groups. The non-normally distributed measurement data were described as median and quartiles [M (Q_1_, Q_3_)], and the Mann-Whitney U rank sum test was used for comparison between groups. Enumeration data were described by n (%). Chi-square test was used for comparison between groups. All the data were subjected to a weighted manner. The masked variance unit pseudo-stratum was sdmvstra, and the masked variance unit pseudo-primary sampling units was sdmvpsu. The confidence interval (CI) was applied for evaluating the reliability of an estimate. A set of weights WTDRD1 was used when an analysis uses the Day 1 dietary recall data (either alone or when Day 1 nutrient data are used in conjunction with MEC data). The set of weights WTDRD1 is applicable to the respondents with Day 1 data. Day 1 weights were constructed by taking the MEC sample weights (WTMEC2YR) and further adjusting for the additional non-response and the differential allocation by day of the week for the dietary intake data collection. The prevalence and treatment rate trends of osteoporosis in postmenopausal women were analyzed using Mann-Kendall trend test. The Z value >0 and *P*<0.05 indicated an increased trend while the Z value >0 and *P*<0.05 represented a decreased trend. Subgroup analysis was conducted in different age, race, BMI, diabetes, hypertension, or glucocorticoid use groups. All statistical tests were performed by a two-sided test with α = 0.05. SAS 9.4 (SAS Institute Inc., Cary, NC, USA) was used to calculate weighted prevalence and treatment rates. Prevalence and treatment rates of osteoporosis were plotted using R 4.0.3 (Institute for Statistics and Mathematics, Vienna, Austria).

## Results

### Comparisons of characteristics between participants with and without physician diagnosed osteoporosis

**Fig 1:**
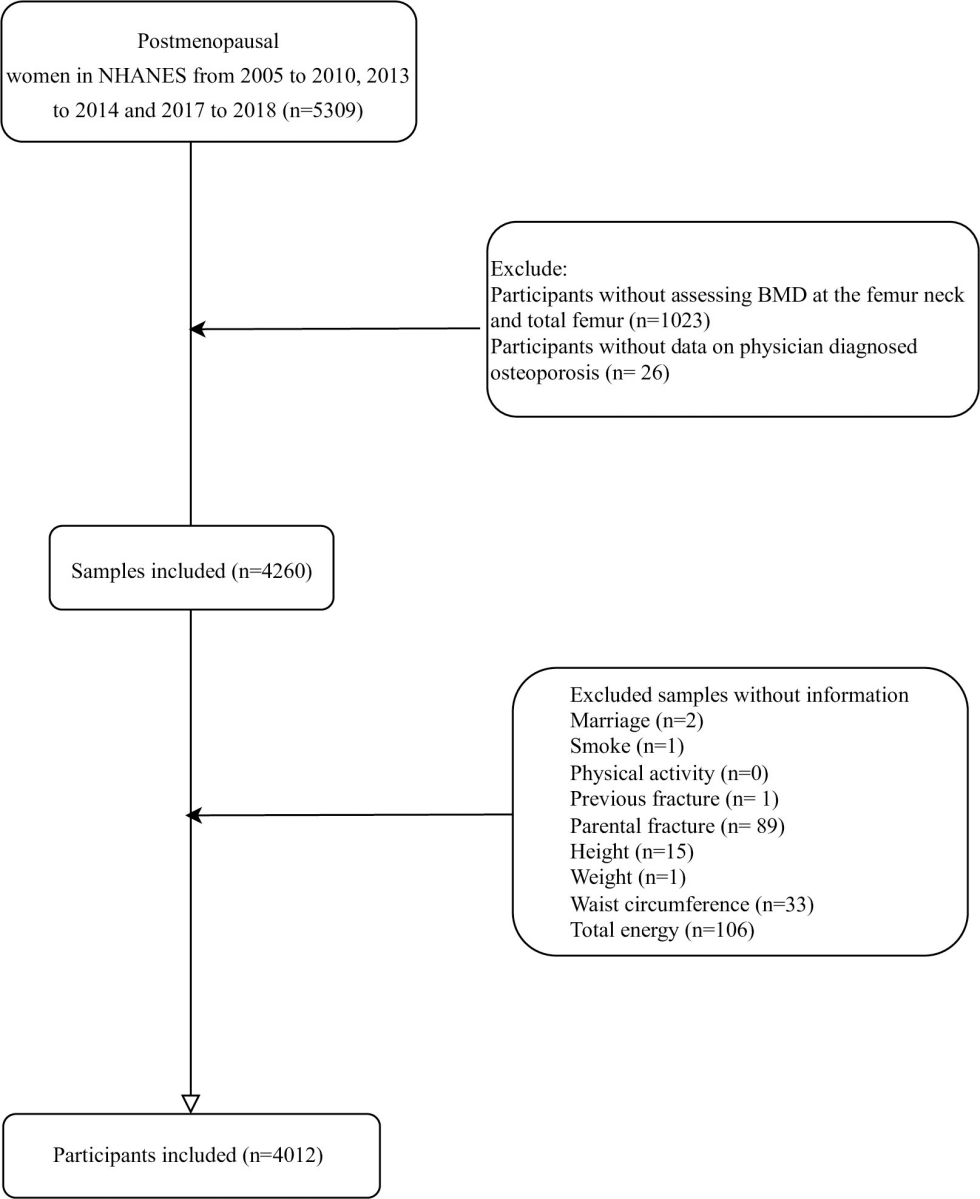
The screen process of participants in the present study.

The data of 5309 postmenopausal women from NHANES from 2005 to 2010, 2013 to 2014 and 2017 to 2018. Participants without data on the assessment of BMD at the femur neck and total femur (n = 1023), without data on physician diagnosed osteoporosis or not (n = 26), without data on marriage (n = 2), smoking (n = 1), previous fracture (n = 1), parental fracture (n = 89), height (n = 15), weight (n = 1), waist circumference (n = 33) or total energy (n = 106) were excluded. Finally, 4012 subjects were included. Among them, 715 people had physician diagnosed osteoporosis and 3297 people did not self-report to have osteoporosis (Fig 1). There were 414 participants diagnosed with osteoporosis through BMD, and 1982 subjects diagnosed with osteopenia.

The percentage of smokers (45.71% vs 42.62%), previous fracture (4.26% vs 1.01%), glucocorticoid use (14.44% vs 8.49%), and complicated with hypertension (64.52% vs 56.19%) in the physician diagnosed osteoporosis group was higher than non-physician diagnosed osteoporosis group. The mean physical activity (1318.44 MET·min/week vs 1875.40 MET·min/week) in the physician diagnosed osteoporosis group was lower than non-physician diagnosed osteoporosis group (Table 1).

**Table 1:** Comparisons of characteristics between participants with and without physician diagnosed osteoporosis.

Variables	Total (n = 4012)	Physician diagnosed osteoporosis (n = 715)	Non-physician diagnosed osteoporosis (n = 3297)	Statistical magnitude	*P*
Age, years, Mean (S.E)	62.48 (0.25)	67.15 (0.50)	61.50 (0.28)	t = 9.94	<0.001
Race, n(%)				χ^2^ = 20.58	<0.001
Non-Hispanic White	2048 (76.95)	443 (82.77)	1605 (75.72)		
Non-Hispanic Black	727 (8.68)	69 (4.49)	658 (9.56)		
Other	1237 (14.38)	203 (12.73)	1034 (14.73)		
Education, n (%)				χ^2^ = 4.31	0.365
Less Than 9th Grade	506 (5.84)	91 (5.92)	415 (5.82)		
9-11th Grade	562 (10.73)	92 (12.34)	470 (10.39)		
High School Grad/GED or Equivalent	1029 (27.45)	202 (29.55)	827 (27.00)		
Some College or AA degree	1113 (29.66)	182 (26.22)	931 (30.38)		
College Graduate or above	797 (26.33)	146 (25.97)	651 (26.41)		
Marriage, n(%)				χ^2^ = 40.52	<0.001
Married	2002 (57.21)	347 (53.79)	1655 (57.93)		
Widowed	843 (16.92)	202 (25.67)	641 (15.07)		
Divorced	684 (17.09)	97 (13.22)	587 (17.90)		
Separated	130 (1.73)	25 (2.25)	105 (1.62)		
Never married	255 (4.80)	30 (2.98)	225 (5.18)		
Living with partner	98 (2.26)	14 (2.10)	84 (2.29)		
PIR, Mean (S.E)	3.21 (0.05)	2.95 (0.10)	3.26 (0.05)	t = -3.08	0.003
Drink, n (%)				χ^2^ = 4.44	0.035
No	1680 (33.58)	318 (37.02)	1362 (32.86)		
Yes	2237 (66.42)	378 (62.98)	1859 (67.14)		
Smoke, n (%)				χ^2^ = 1.35	0.246
No	2409 (56.84)	405 (54.29)	2004 (57.38)		
Yes	1603 (43.16)	310 (45.71)	1293 (42.62)		
Physical activity, MET·min/week, Mean (S.E)	1778.41 (81.20)	1318.44 (141.18)	1875.40 (94.24)	t = -3.25	0.002
Hypertension, n (%)				χ^2^ = 7.50	0.006
No	1483 (42.36)	218 (35.48)	1265 (43.81)		
Yes	2529 (57.64)	497 (64.52)	2032 (56.19)		
Diabetes, n (%)				χ^2^ = 0.52	0.471
No	3113 (82.99)	569 (84.25)	2544 (82.72)		
Yes	899 (17.01)	146 (15.75)	753 (17.28)		
Previous fracture, n (%)				χ^2^ = 27.19	<0.001
No	3943 (98.43)	682 (95.74)	3261 (98.99)		
Yes	69 (1.57)	33 (4.26)	36 (1.01)		
Parental fracture, n (%)				χ^2^ = 4.57	0.032
No	3561 (87.08)	614 (83.57)	2947 (87.82)		
Yes	451 (12.92)	101 (16.43)	350 (12.18)		
Glucocorticoid use, n (%)				χ^2^ = 9.25	0.002
No	3676 (90.47)	612 (85.56)	3064 (91.51)		
Yes	336 (9.53)	103 (14.44)	233 (8.49)		
Anti-osteoporosis therapy, n (%)				χ^2^ = 1140.85	<0.001
No	3427 (85.95)	212 (29.51)	3215 (97.85)		
Yes	585 (14.05)	503 (70.49)	82 (2.15)		
BMI, kg/m^2^, Mean (S.E)	28.47 (0.15)	27.17 (0.38)	28.74 (0.15)	t = -3.98	<0.001
BMI, n(%)				χ^2^ = 12.67	<0.001
<25 kg/m^2^	1163 (31.63)	266 (41.03)	897 (29.65)		
≥25 kg/m^2^	2849 (68.37)	449 (58.97)	2400 (70.35)		
Waist circumference, cm, Mean (S.E)	96.73 (0.38)	94.12 (1.02)	97.28 (0.37)	t = -3.00	0.004
Cotinine, ng/ml, Mean (S.E)	41.39 (2.90)	44.20 (6.57)	40.80 (2.77)	t = 0.55	0.582
Cadmium, ug/L, Mean (S.E)	0.57 (0.02)	0.61 (0.03)	0.56 (0.02)	t = 1.43	0.157
Lead, ug/dL, Mean (S.E)	1.67 (0.02)	1.58 (0.04)	1.69 (0.03)	t = -2.35	0.021
Iron, umol/L, Mean (S.E)	14.77 (0.15)	14.76 (0.32)	14.77 (0.16)	t = -0.03	0.973
Mercury, umol/L, Mean (S.E)	8.17 (0.29)	6.74 (0.36)	8.47 (0.31)	t = -4.40	<0.001
25[OH]D, nmol/L, Mean (S.E)	74.59 (0.95)	80.13 (1.63)	73.40 (0.96)	t = 4.49	<0.001
Total energy, kcal, Mean (S.E)	1708.55 (16.73)	1646.95 (34.56)	1721.54 (18.41)	t = -1.97	0.053
Day 1 calcium intake, mg, Mean (S.E)	834.41 (11.35)	808.83 (19.21)	839.80 (13.15)	t = -1.33	0.188
Calcium intake in dietary supplement, n (%)				χ^2^ = 13.93	< .001
Yes	1493 (39.44)	353 (48.26)	1140 (37.58)		
Unknown	2519 (60.56)	362 (51.74)	2157 (62.42)		
Total calcium intake, mg, Mean (S.E)	1116.74 (17.02)	1199.01 (30.93)	1099.39 (18.67)	t = 2.94	0.004
Day 1 vitamin D intake, n (%)				χ^2^ = 0.60	0.741
No	55 (1.18)	12 (1.47)	43 (1.11)		
Yes	3263 (78.05)	584 (78.43)	2679 (77.97)		
Unknown	694 (20.77)	119 (20.09)	575 (20.91)		
Vitamin D intake in dietary supplement, n (%)				χ^2^ = 9.86	0.002
Yes	1451 (39.05)	337 (46.83)	1114 (37.41)		
Unknown	2561 (60.95)	378 (53.17)	2183 (62.59)		

S.E: standard error, GED: General Equivalent Diploma, PIR: poverty-to-income ratio, BMI: body mass index, 25[OH]D: 25-hydroxycholecalcifer-ol

### Comparisons of characteristics among participants with osteoporosis or osteopenia diagnosed by BMD and normal people

The mean age (70.67 years vs 63.75 years vs 58.85 years), BMI (24.60 kg/m^2^ vs 27.10 kg/m^2^ vs 31.20 kg/m^2^) and waist circumference (88.75 cm vs 93.81 cm vs 102.51 cm) were statistical difference among participants with osteoporosis or osteopenia diagnosed by BMD and normal people. The percentages of previous fracture (5.91% vs 1.26% vs 0.96%) and glucocorticoid use (11.29% vs 9.09% vs 9.70%) were statistical difference among participants with osteoporosis or osteopenia diagnosed by BMD and normal people (Table 2).

**Table 2:** Comparisons of characteristics among participants with osteoporosis or osteopenia diagnosed by BMD and normal people.

Variables	Total (n = 4012)	Normal bone mass (n = 1616)	Osteopenia (n = 1982)	Osteoporosis (n = 414)	Statistical magnitude	*P*
Age, years, Mean (S.E)	62.48 (0.25)	58.85 (0.33)	63.75 (0.31)	70.67 (0.67)	F = 177.02	<0.001
Race, n(%)					χ^2^ = 120.45	<0.001
Non-Hispanic White	2048 (76.95)	679 (71.11)	1105 (80.32)	264 (82.61)		
Non-Hispanic Black	727 (8.68)	467 (15.02)	225 (4.83)	35 (3.58)		
Other	1237 (14.38)	470 (13.87)	652 (14.86)	115 (13.82)		
Education, n (%)					χ^2^ = 17.07	0.029
Less Than 9th Grade	506 (5.84)	193 (5.63)	242 (5.28)	71 (9.88)		
9-11th Grade	562 (10.73)	215 (9.70)	280 (10.93)	67 (13.91)		
High School Grad/GED or Equivalent	1029 (27.45)	404 (26.52)	517 (28.30)	108 (26.60)		
Some College or AA degree	1113 (29.66)	478 (30.83)	533 (28.45)	102 (31.49)		
College Graduate or above	797 (26.33)	326 (27.32)	406 (27.04)	65 (18.12)		
Marriage, n(%)					χ^2^ = 106.40	<0.001
Married	2002 (57.21)	859 (61.05)	985 (56.90)	158 (42.78)		
Widowed	843 (16.92)	248 (11.33)	439 (17.52)	156 (37.16)		
Divorced	684 (17.09)	281 (16.94)	344 (17.56)	59 (15.04)		
Separated	130 (1.73)	66 (2.06)	54 (1.59)	10 (1.08)		
Never married	255 (4.80)	121 (5.90)	108 (4.26)	26 (3.17)		
Living with partner	98 (2.26)	41 (2.71)	52 (2.18)	5 (0.77)		
PIR, Mean (S.E)	3.21 (0.05)	3.34 (0.07)	3.21 (0.07)	2.61 (0.15)	F = 11.03	<0.001
Drink, n (%)					χ^2^ = 13.03	0.001
No	1680 (33.58)	657 (31.75)	809 (32.90)	214 (45.16)		
Yes	2237 (66.42)	931 (68.25)	1120 (67.10)	186 (54.84)		
Smoke, n (%)					χ^2^ = 0.80	0.671
No	2409 (56.84)	957 (55.71)	1200 (57.68)	252 (56.87)		
Yes	1603 (43.16)	659 (44.29)	782 (42.32)	162 (43.13)		
Physical activity, MET·min/week, Mean (S.E)	1778.41 (81.20)	1966.07 (130.84)	1761.29 (108.35)	1082.53 (131.71)	F = 11.93	<0.001
Hypertension, n (%)					χ^2^ = 10.33	0.006
No	1483 (42.36)	599 (41.12)	749 (44.79)	135 (33.86)		
Yes	2529 (57.64)	1017 (58.88)	1233 (55.21)	279 (66.14)		
Diabetes, n (%)					χ^2^ = 27.12	<0.001
No	3113 (82.99)	1183 (77.57)	1586 (86.46)	344 (86.35)		
Yes	899 (17.01)	433 (22.43)	396 (13.54)	70 (13.65)		
Previous fracture, n (%)					χ^2^ = 37.88	<0.001
No	3943 (98.43)	1601 (99.04)	1951 (98.74)	391 (94.09)		
Yes	69 (1.57)	15 (0.96)	31 (1.26)	23 (5.91)		
Parental fracture, n (%)					χ^2^ = 6.64	0.036
No	3561 (87.08)	1469 (88.86)	1740 (86.83)	352 (80.99)		
Yes	451 (12.92)	147 (11.14)	242 (13.17)	62 (19.01)		
Glucocorticoid use, n (%)					χ^2^ = 0.78	0.679
No	3676 (90.47)	1487 (90.30)	1811 (90.91)	378 (88.71)		
Yes	336 (9.53)	129 (9.70)	171 (9.09)	36 (11.29)		
Anti-osteoporosis therapy, n (%)					χ^2^ = 211.78	<0.001
No	3427 (85.95)	1528 (95.69)	1627 (83.07)	272 (61.05)		
Yes	585 (14.05)	88 (4.31)	355 (16.93)	142 (38.95)		
Body mass index, kg/m^2^, Mean (S.E)	28.47 (0.15)	31.20 (0.22)	27.10 (0.17)	24.60 (0.44)	F = 145.58	<0.001
Body mass index, n(%)					χ^2^ = 172.48	<0.001
Body Mass Index<25 kg/m^2^	1163 (31.63)	226 (15.59)	688 (38.88)	249 (58.60)		
Body Mass Index≥25 kg/m^2^	2849 (68.37)	1390 (84.41)	1294 (61.12)	165 (41.40)		
Waist circumference, cm, Mean (S.E)	96.73 (0.38)	102.51 (0.51)	93.81 (0.48)	88.75 (1.16)	F = 92.96	<0.001
Cotinine, ng/ml, Mean (S.E)	41.39 (2.90)	39.75 (4.18)	38.94 (3.29)	62.03 (8.16)	F = 3.93	0.024
Cadmium, ug/L, Mean (S.E)	0.57 (0.02)	0.54 (0.03)	0.56 (0.02)	0.72 (0.04)	F = 8.41	<0.001
Lead, ug/dL, Mean (S.E)	1.67 (0.02)	1.58 (0.03)	1.72 (0.03)	1.79 (0.06)	F = 8.26	<0.001
Iron, umol/L, Mean (S.E)	14.77 (0.15)	14.51 (0.22)	14.87 (0.18)	15.28 (0.41)	F = 1.61	0.207
Mercury, umol/L, Mean (S.E)	8.17 (0.29)	8.62 (0.48)	8.26 (0.36)	5.65 (0.42)	F = 15.19	<0.001
25[OH]D, nmol/L, Mean (S.E)	74.59 (0.95)	71.58 (1.26)	76.78 (1.19)	74.86 (2.29)	F = 5.83	0.004
Total energy, kcal, Mean (S.E)	1708.55 (16.73)	1752.45 (23.69)	1686.69 (24.72)	1646.28 (41.81)	F = 3.87	0.025
Day 1 calcium intake, mg, Mean (S.E)	834.41 (11.35)	841.31 (16.01)	836.44 (16.88)	793.82 (30.14)	F = 1.03	0.361
Calcium intake in dietary supplement, n (%)					χ^2^ = 5.99	0.050
Yes	1493 (39.44)	535 (36.23)	795 (41.44)	163 (41.71)		
Unknown	2519 (60.56)	1081 (63.77)	1187 (58.56)	251 (58.29)		
Total calcium intake, mg, Mean (S.E)	1116.74 (17.02)	1091.76 (25.84)	1139.14 (23.30)	1096.13 (42.26)	F = 1.02	0.365
Day 1 vitamin D intake, n (%)					χ^2^ = 11.77	0.019
No	55 (1.18)	25 (1.23)	22 (0.94)	8 (2.27)		
Yes	3263 (78.05)	1294 (76.44)	1644 (80.10)	325 (73.38)		
Unknown	694 (20.77)	297 (22.33)	316 (18.96)	81 (24.35)		
Vitamin D intake in dietary supplement, n (%)					χ^2^ = 6.88	0.032
Yes	1451 (39.05)	523 (35.33)	762 (41.32)	166 (41.96)		
Unknown	2561 (60.95)	1093 (64.67)	1220 (58.68)	248 (58.04)		

BMD: bone mineral density, S.E: standard error, GED: General Equivalent Diploma, PIR: poverty-to-income ratio, 25[OH]D: 25-hydroxycholecalcifer-ol

### The prevalence of osteoporosis in postmenopausal women during the five cycles

**Fig 2:**
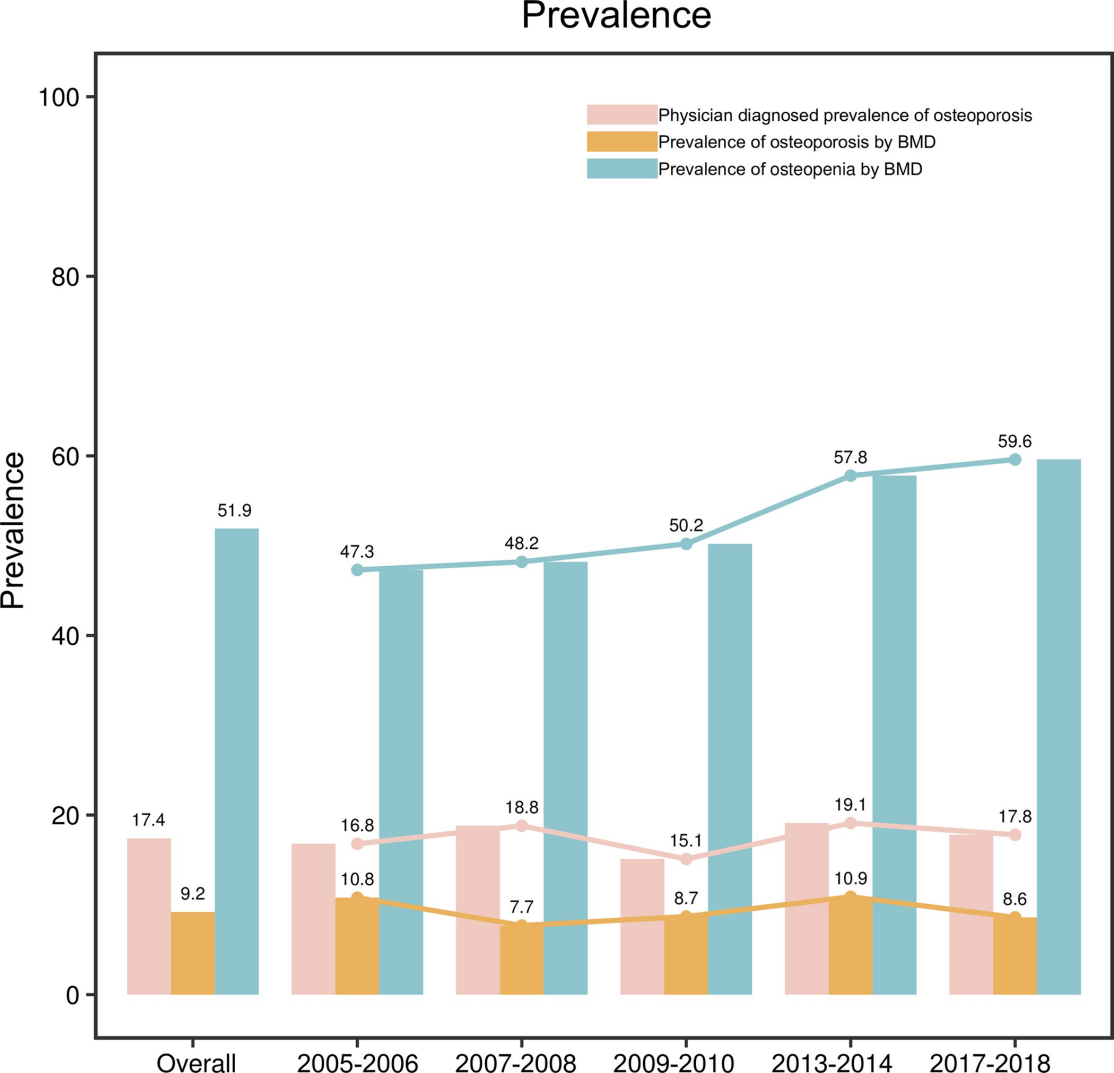
The prevalence of osteoporosis and osteopenia in postmenopausal women.

The overall prevalence of physician diagnosed osteoporosis was 17.4% in 2005–2006, 2007–2008, 2009–2010, 2013–2014, and 2017–2018. From 2005 to 2018, the prevalence of physician diagnosed osteoporosis was fluctuated in a small range and remained relatively stable within a certain range (Mann-Kendall trend test: Z = 2.20, *P* = 0.027). The prevalence of osteoporosis in postmenopausal women determined by BMD examination reached 9.2% during the five cycles. From 2005 to 2018, the prevalence of physician diagnosed osteoporosis fluctuated in a small range. For osteopenia measured by BMD, the prevalence was 59.6% and a gradual increasing trend was found between 2005 and 2018 (Mann-Kendall trend test: Z = 2.20, *P* = 0.027) (Fig 2).

### The treatment rate of osteoporosis in postmenopausal women during the five cycles

**Fig 3:**
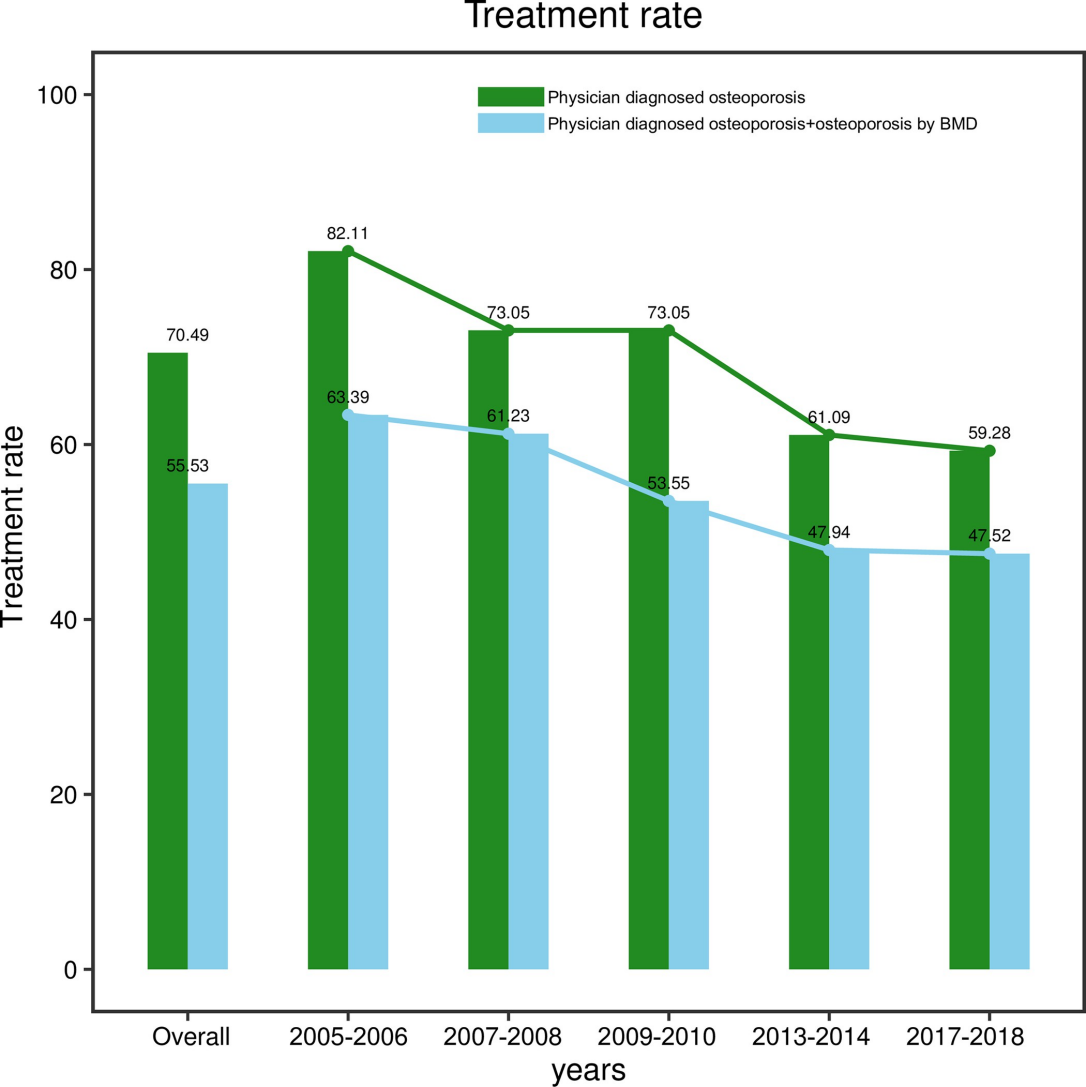
The treatment rate of osteoporosis in postmenopausal women.

Among patients with physician diagnosed osteoporosis, the treatment rate reached 70.49%. The treatment rate decreased from 2005 to 2008, and further decreased from 2009 to 2018 (Mann-Kendall trend test: Z = -2.20, *P* = 0.027). In 2018, the treatment rate of these patients was 59.28%. In order to avoid overestimating the treatment rate of osteoporosis, the actual treatment rate of osteoporosis was calculated. The actual treatment rate of osteoporosis patients was 55.53%. During 2005–2018, the actual treatment rate of osteoporosis showed a continuous decline (Mann-Kendall trend test: Z = -2.20, *P* = 0.027), and dropped to 47.52% in 2018 (Fig 3).

### Subgroup analysis of the prevalence of osteoporosis and its treatment rate in postmenopausal women during the five cycles

The number of samples during the five cycles analyzed in each subgroup was shown Table 3.

**Table 3:** The number of samples during the five cycles analyzed in each subgroup.

Variables	Total (n = 4012)	year0506 (n = 694)	year0708 (n = 1095)	year0910 (n = 1058)	year1314 (n = 598)	year1718 (n = 567)
Age, n (%)						
Age<65years old	2199 (60.11)	371 (58.41)	587 (62.43)	584 (62.00)	334 (59.29)	323 (57.05)
Age≥65years old	1813 (39.89)	323 (41.59)	508 (37.57)	474 (38.00)	264 (40.71)	244 (42.95)
Race, n (%)						
Non-Hispanic White	2048 (76.95)	415 (83.02)	575 (77.44)	574 (76.65)	282 (74.54)	202 (71.32)
Non-Hispanic Black	727 (8.68)	142 (8.67)	213 (9.62)	160 (9.40)	92 (7.43)	120 (7.49)
Other	1237 (14.38)	137 (8.31)	307 (12.94)	324 (13.95)	224 (18.03)	245 (21.19)
BMI, n (%)						
<25 kg/m^2^	1163 (31.63)	228 (34.03)	318 (32.64)	264 (28.69)	197 (36.17)	156 (27.03)
≥25 kg/m^2^	2849 (68.37)	466 (65.97)	777 (67.36)	794 (71.31)	401 (63.83)	411 (72.97)
Hypertension, n (%)						
No	1483 (42.36)	263 (38.58)	393 (43.01)	382 (42.35)	237 (44.10)	208 (44.52)
Yes	2529 (57.64)	431 (61.42)	702 (56.99)	676 (57.65)	361 (55.90)	359 (55.48)
Diabetes, n (%)						
No	3113 (82.99)	561 (86.11)	859 (84.10)	807 (83.38)	459 (81.85)	427 (78.02)
Yes	899 (17.01)	133 (13.89)	236 (15.90)	251 (16.62)	139 (18.15)	140 (21.98)
Glucocorticoid use, n (%)						
No	3676 (90.47)	632 (89.54)	1018 (91.45)	952 (89.50)	553 (92.28)	521 (89.89)
Yes	336 (9.53)	62 (10.46)	77 (8.55)	106 (10.50)	45 (7.72)	46 (10.11)
Physician diagnosed osteoporosis, n (%)						
No	3297 (82.59)	575 (83.15)	885 (81.17)	884 (84.91)	490 (80.93)	463 (82.20)
Yes	715 (17.41)	119 (16.85)	210 (18.83)	174 (15.09)	108 (19.07)	104 (17.80)
Bone mass status by BMD, n (%)						
Normal	1616 (38.91)	297 (41.85)	482 (44.13)	439 (41.11)	200 (31.31)	198 (31.88)
Osteoporosis	414 (9.22)	81 (10.81)	99 (7.66)	103 (8.71)	69 (10.91)	62 (8.57)
Osteopenia	1982 (51.87)	316 (47.35)	514 (48.22)	516 (50.18)	329 (57.77)	307 (59.55)
Anti-osteoporosis therapy, n (%)						
No	3427 (85.95)	577 (83.18)	908 (83.44)	915 (87.91)	526 (87.86)	501 (88.44)
Yes	585 (14.05)	117 (16.82)	187 (16.56)	143 (12.09)	72 (12.14)	66 (11.56)

BMI: body mass index, BMD: bone mineral density

### Age

**Fig 4:**
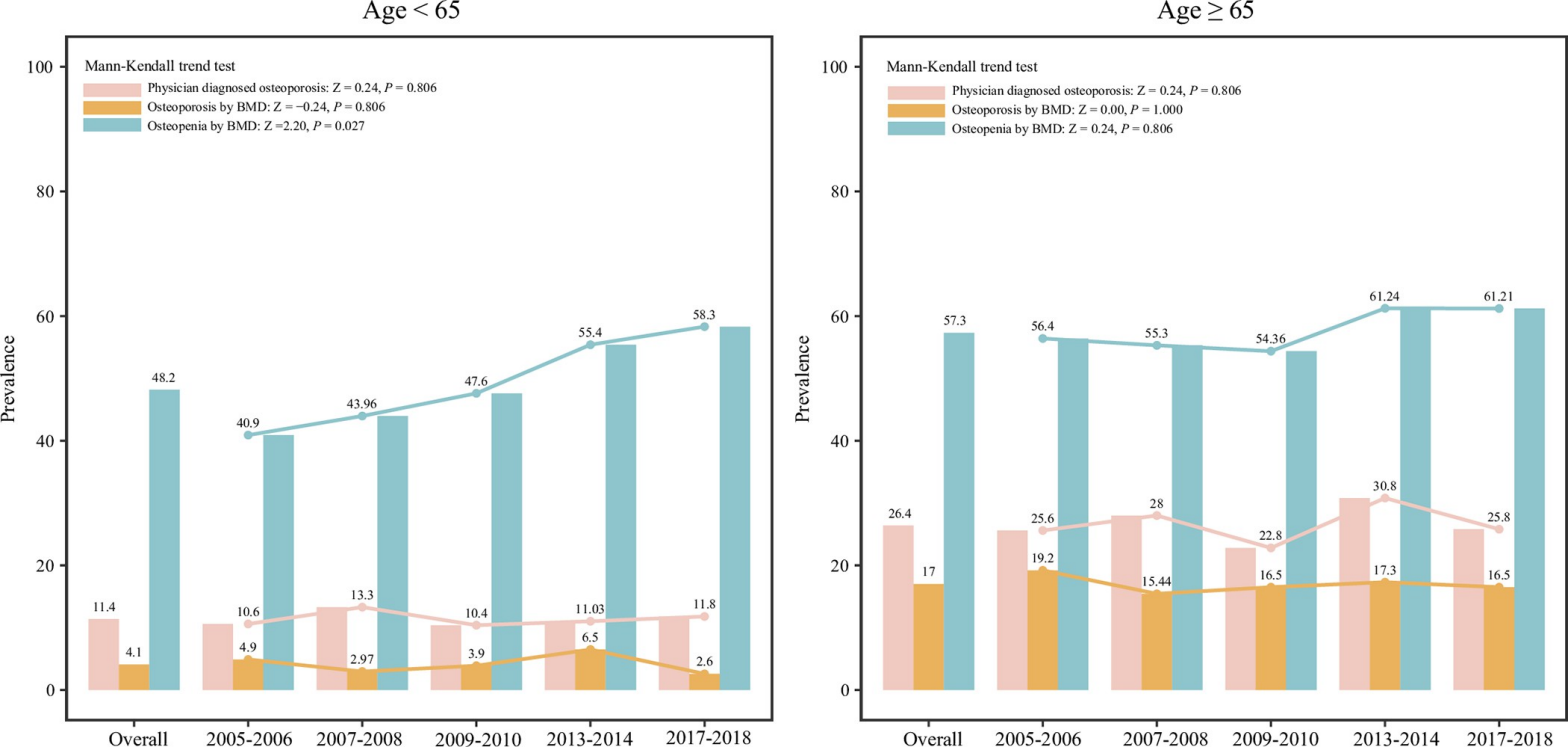
The prevalence of osteoporosis and osteopenia in postmenopausal women from different age groups.

In people <65 years, the prevalence of physician diagnosed osteoporosis, and osteoporosis or osteopenia measured by BMD was 11.4%, 4.1% and 48.2%, respectively. The prevalence of osteopenia increased during 2005–2018 (Mann-Kendall trend test: Z = 2.20, *P* = 0.027). In the subgroup of people aged ≥65 years, the overall prevalence of physician diagnosed osteoporosis and osteoporosis or osteopenia measured by BMD were 26.4%, 17% and 57.3%, respectively (Fig 4).

### Race

**Fig 5:**
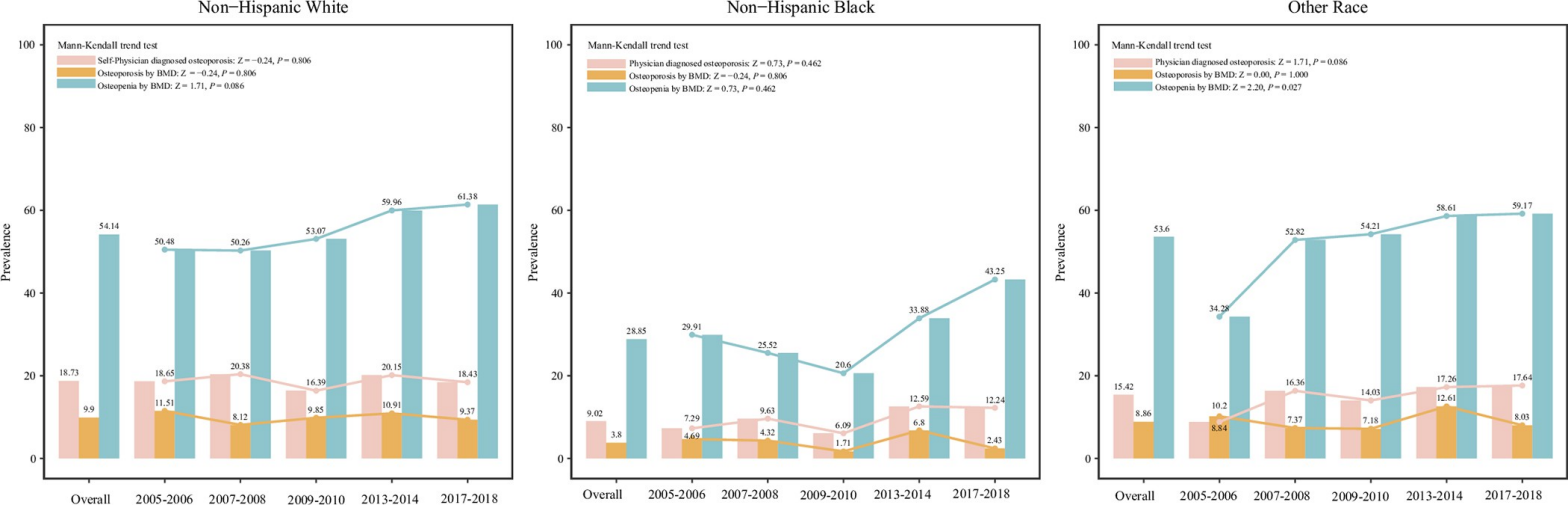
The prevalence of osteoporosis and osteopenia in postmenopausal women from different race groups.

In the non-Hispanic White group, the overall prevalence of physician diagnosed osteoporosis was 18.73%, the overall prevalence of osteoporosis or osteopenia measured by BMD were 9.9% and 54.14%, respectively. The prevalence presented a relatively stable trend over the 5 cycles. In non-Hispanic Black group, the overall prevalence of physician diagnosed osteoporosis and osteoporosis or osteopenia measured by BMD were 9.02%, 3.8% and 28.85%, respectively. The prevalence of osteopenia decreased slowly during 2005–2010 and then increased rapidly to 2018. The prevalence of osteoporosis measured by BMD or physician diagnosed was generally stable. The prevalence of osteopenia increased year by year over the 5 cycles (Mann-Kendall trend test: Z = 2.20, *P* = 0.027), while the prevalence of physician diagnosed and BMD-determined osteoporosis remained relatively stable (Fig 5).

### BMI

**Fig 6:**
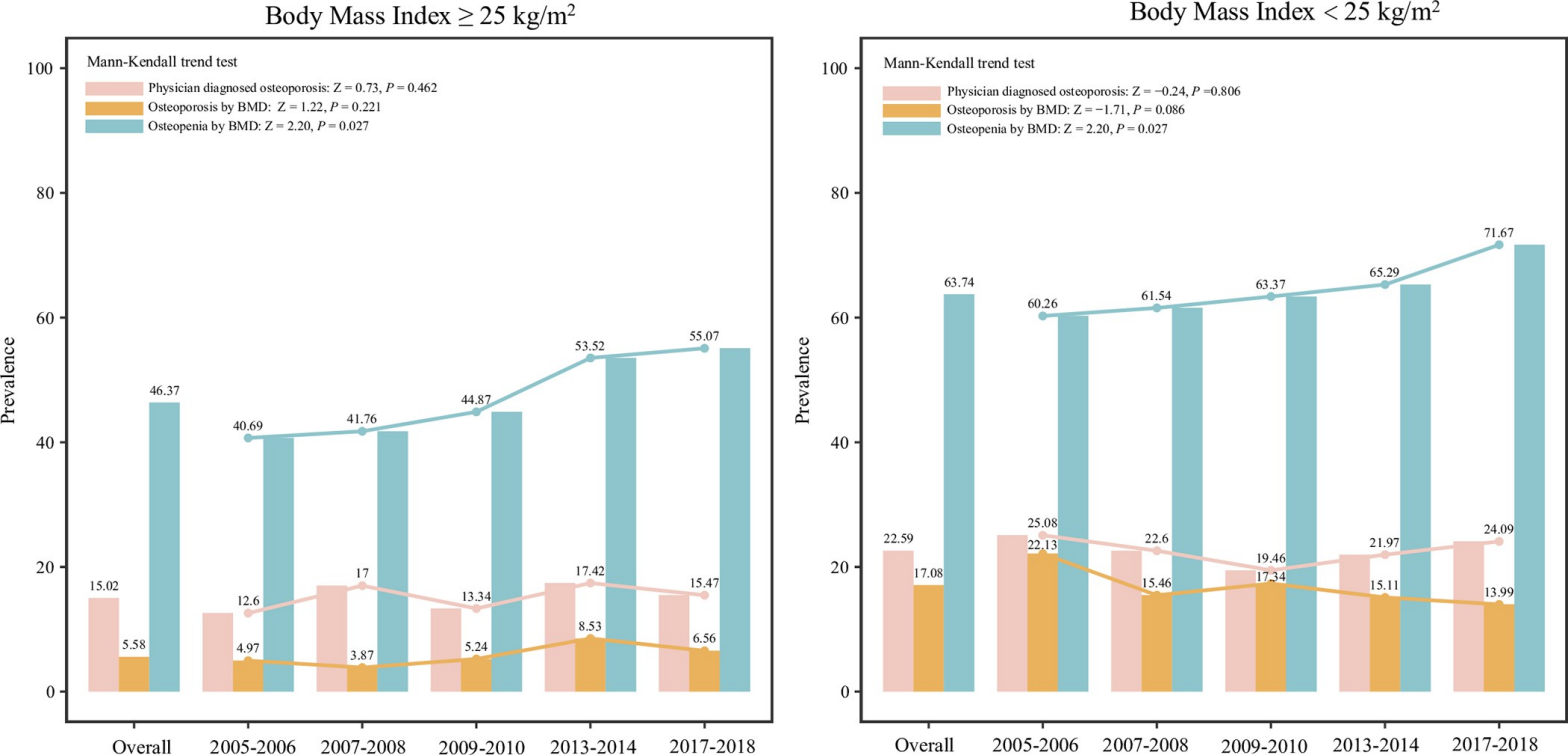
The prevalence of osteoporosis and osteopenia in postmenopausal women from different BMI groups.

In BMI≥25kg/m^2^ group, the overall prevalence of physician diagnosed osteoporosis and osteoporosis or osteopenia diagnosed by BMD were 15.02%, 5.58% and 46.37%, respectively. The prevalence of osteopenia was elevated during the five cycles (Mann-Kendall trend test: Z = 2.20, *P* = 0.027), and the prevalence of physician diagnosed osteoporosis and osteoporosis diagnosed by BMD exhibited a relatively stable fluctuation. In BMI<25kg/m^2^ group, the prevalence of physician diagnosed osteoporosis was 22.59%. The overall prevalence of osteoporosis diagnosed by BMD was 17.08%, and a relatively stable fluctuation tread was observed during 2005–2018. The overall prevalence of osteopenia diagnosed by BMD was 63.74% and the prevalence trend was increased from 2005–2018 (Mann-Kendall trend test: Z = 2.20, *P* = 0.027) (Fig 6).

### Diabetes

**Fig 7:**
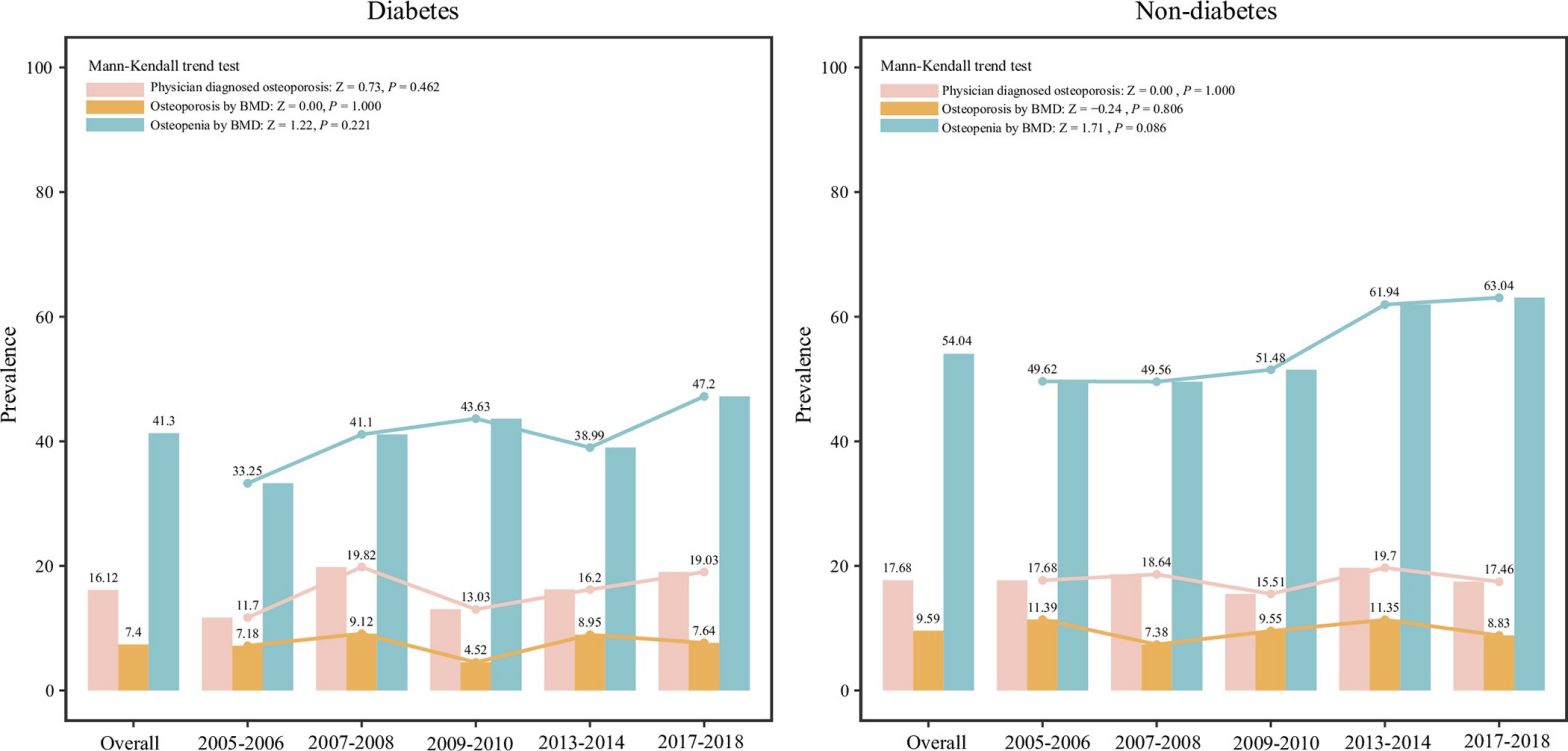
The prevalence of osteoporosis and osteopenia in postmenopausal women with or without diabetes.

In subjects with diabetes, the overall prevalence of physician diagnosed osteoporosis and osteoporosis or osteopenia measured by BMD were 16.12%, 7.4% and 41.3%, respectively. The prevalence of physician diagnosed osteoporosis and osteoporosis or osteopenia showed relative stable fluctuations from 2005–2018. In those without diabetes, the overall prevalence of physician diagnosed osteoporosis and osteoporosis or osteopenia measured by BMD 17.68%, 9.59% and 54.04%, respectively. Relatively stationary trends were found in the prevalence of physician diagnosed osteoporosis and osteoporosis or osteopenia measured by BMD during 2005–2018 (Fig 7).

### Hypertension

**Fig 8:**
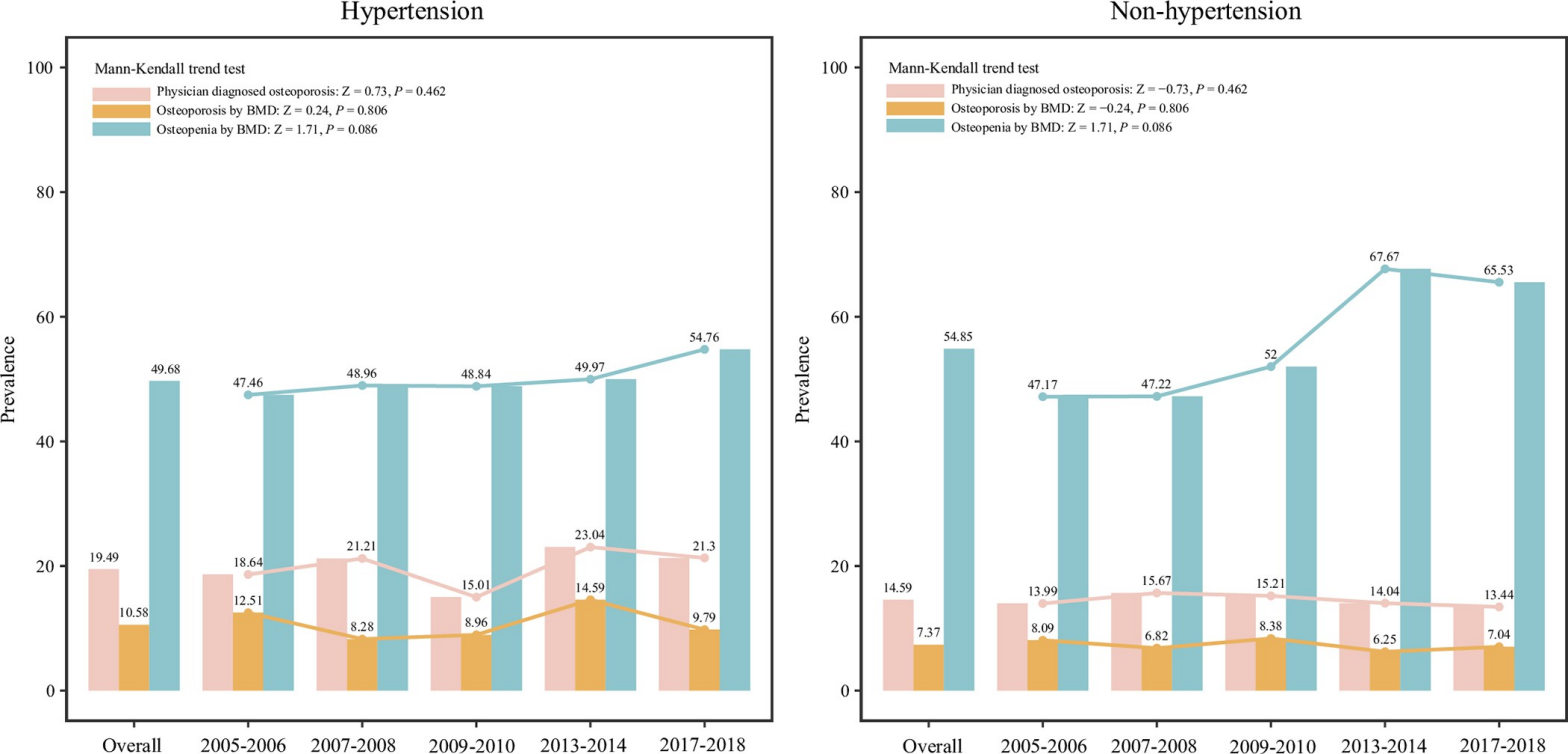
The prevalence of osteoporosis and osteopenia in postmenopausal women with or without hypertension.

In people with hypertension, the overall prevalence of physician diagnosed, BMD-measured osteoporosis and osteopenia were 19.49%, 10.58% and 49.68%, respectively, and presented relative stable fluctuations during 2005–2018. The overall prevalence of physician diagnosed, BMD-measured osteoporosis and osteopenia were 14.59%, 7.37% and 54.85% in those without hypertension. Relative stable fluctuations were found in the prevalence of physician diagnosed, BMD-measured osteoporosis and osteopenia during 2005–2018 in those without hypertension (Fig 8).

### Glucocorticoid use

**Fig 9:**
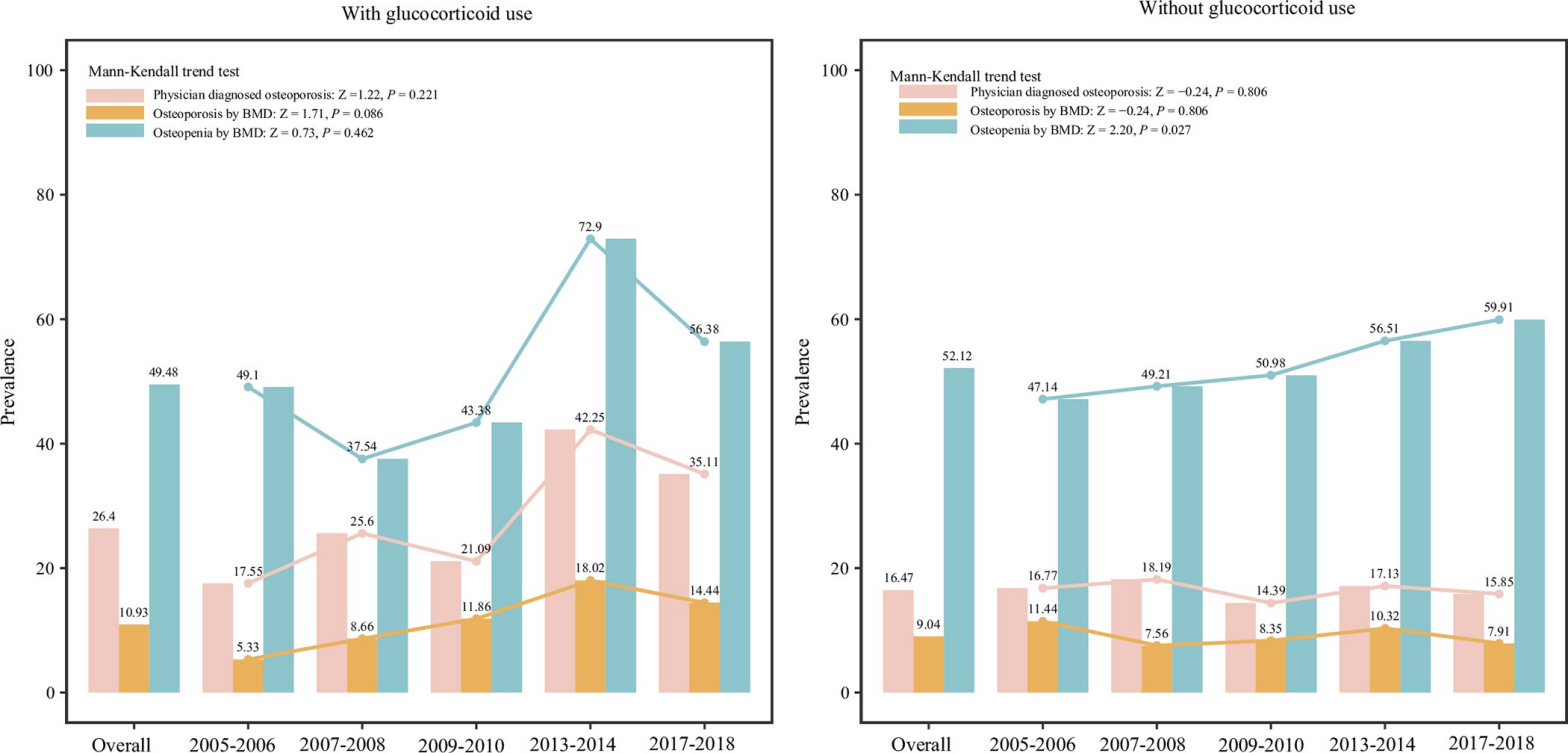
The prevalence of osteoporosis and osteopenia in postmenopausal women with or without glucocorticoid use.

In subjects used glucocorticoid, the overall prevalence of physician diagnosed osteoporosis, osteoporosis and osteopenia were 26.4%, 10.93% and 49.48%, respectively. The prevalence of physician diagnosed osteoporosis was decreased from 2005–2008, increased from 2009–2014 and then decreased to 2018. The prevalence of osteoporosis diagnosed by BMD was increased during 2005–2014, and decreased from 2014–2018. The prevalence of osteopenia was decreased from 2005 to 2008, and increased from 2009–2014. The overall prevalence of physician diagnosed, BMD-measured osteoporosis and osteopenia were 16.47%, 9.04% and 52.12%, respectively in those without glucocorticoid use. The prevalence of osteopenia showed an increase during the 5 cycles (Mann-Kendall trend test: Z = 2.20, *P* = 0.027), while the prevalence of physician diagnosed and BMD-detected osteoporosis showed relatively stable fluctuations (Fig 9).

## Discussion

The current study evaluated the trends of prevalence and treatment rate of osteoporosis in postmenopausal women from 2005–2018. The results depicted that the overall prevalence of physician diagnosed osteoporosis in postmenopausal women was 17.4% in 2005–2006, 2007–2008, 2009–2010, 2013–2014, and 2017–2018. The treatment rate of these patients was 70.49%. The prevalence of osteoporosis determined by BMD examination in postmenopausal women was 9.2% during the five cycles and the actual treatment rate of these patients was 55.53%. For osteopenia measured by BMD, the prevalence was 59.6%. In people <65 years, the prevalence of physician diagnosed osteoporosis, and osteoporosis or osteopenia measured by BMD was 11.4%, 4.1% and 48.2%, respectively. The prevalence of osteopenia was increased during 2005–2018. The findings might provide a reference for the management of osteoporosis or osteopenia in postmenopausal women

In the present study, the overall prevalence of physician diagnosed osteoporosis was 17.4% and osteoporosis diagnosed by BMD was 9.2%. The results were similar to some studies. Baccaro et al. found that the prevalence of osteoporosis based on self-reporting was 21.3%, and 16.7% of the participants reported that the diagnosis of osteoporosis had been made by bone densitometry in 622 women aged over 50 years [13]. A study based on the data from the National Health Registry between 2012 and 2018 depicted that the overall prevalence of osteoporosis was 2.4% in those over 50 years of age in Colombia [14]. A meta-analysis showed that the prevalence of osteoporosis in postmenopausal females undergoing total knee and hip arthroplasty patients was 38.3% [15]. Xiao et al. conducted a systematic review and meta-analysis, which found that the global prevalence of osteoporosis was 19.7% according to the World Health Organization diagnostic criteria and the prevalence varied greatly in different countries (from 4.1% in Netherlands to 52.0% in Turkey) and continents (from Oceania 8.0% to 26.9% in Africa) [16]. The prevalence of physician diagnosed and DXA-confirmed osteoporosis was 12.7% and 5.9% in females, respectively based on data from community-dwelling older adults from the Canadian Longitudinal Study on Aging [17]. Buttros and cols conducted a cross-sectional study and found a prevalence of 24.6% of osteoporosis in postmenopausal women (aged 40–75 years) using BMD for diagnosis [18]. Some other studies also found a relative high prevalence of osteoporosis in postmenopausal women. Khinda et al. identified that the prevalence of osteoporosis was 30.50% in postmenopausal women of Punjab, India [19]. Another hospital-based study revealed that the prevalence of osteoporosis has significantly increased in postmenopausal women to 58.4% [20]. The lower prevalence of osteoporosis in our study than that reported in some other studies may be due to the difference in study population. Under-diagnosis or under-recording as well as recall bias might exist in the NHANES.

We also found no significant change in the trend of prevalence of osteoporosis in postmenopausal women during 2005–2018, which was supported by a study from Smith et al. The study found no statistically significant trend in reported osteoporosis prevalence over time since at least 2001 [21]. Another study of Liu et al. constructed a prediction model and estimated that 6.6% Chinese elderly were suffering from osteoporosis and the number might increase to 8.2% in 2010, and 13.6% in 2050 [22]. The findings were distinct with this study, which may be because the study included all Chinese elderly population. The incidence of osteoporosis and the risk of fractures increase with age, and postmenopausal women should increase the frequency of detection on osteoporosis via DXA for the early identification of those who at high risk of osteoporosis or those who have already suffer osteoporosis.

In this study, we also observed a prevalence of 59.6% of osteopenia in postmenopausal women and the prevalence of osteopenia in postmenopausal women was increased from 2005 to 2018. The prevalence of osteopenia was reported to be 44.20% in postmenopausal women of India [19], 44.0% in postmenopausal women of South Korea [21], 43.6% in postmenopausal women of Brazil [18]. The increase trend of prevalence of osteopenia in postmenopausal women might due to the awareness of detection. In our study, the treatment rate of osteoporosis patients was decreased during 2005–2018. Previously, McArthur et al. observed that for community-dwelling older adults who have already diagnosed with osteoporosis, 76.8% females were not taking an osteoporosis medication [17]. A systematic review and meta-analysis identified that the treatment rate of osteoporosis in patients undergoing total knee and hip arthroplasty patients was 32.9% [15]. The total treatment frequency for osteoporosis in those aged ≥50 years was 1.28% in Greenland and 4.71% in Denmark, and among people aged ≥80 years, the treatment rates for osteoporosis were 3.41% and 11.18% in Greenland and Denmark, respectively [23]. These data suggested the low treatment rate of osteoporosis in people including postmenopausal women. In postmenopausal women, the awareness and attention of osteoporosis and osteopenia is still insufficient, and the promotion of awareness of bone health needs to be strengthened. Serum 25-hydroxyvitamin D levels should be monitored. The low treatment rate of osteoporosis and osteopenia reminded that more attention should be paid on the treatments of postmenopausal women who already diagnosed with osteoporosis and osteopenia. For women who diagnosed with osteopenia, nutrition supplement such as vitamin D and calcium are necessary, and adequate exercise are needed to prevent the occurrence of osteoporosis. For those diagnosed with osteoporosis, medication treatments combined with nutrition support such as maintaining serum vitamin D sufficiency and calcium supplemented are required. Individualized treatments are needed for women diagnosed with osteoporosis and treatment compliance for osteoporosis needs to be improved [24]. Routinely assessing the adherence with therapy are needed for continued or modified treatment.

In the current study, the prevalence of osteoporosis and osteopenia in women aged ≥65 years was higher than those <65 years. This was supported by previous studies [25]. Tang et al. found the prevalence of osteoporosis in postmenopausal women aged 40–49 years, 50–59 years, 60–69 years, 70–79 years, and ≥80 years were 16.0%, 18.4%, 37.5%, 52.9%, and 68.0% in a total of 5728 postmenopausal women aged ≥40 years [26]. In particular, the prevalence of osteoporosis in women aged ≥65 years was four times that of those <65 years of age. Currently, the guidelines for routine osteoporosis screening and BMD testing in women aged ≥65 years was still not clear. The findings in this study might provide reference for improving the suggestion of routine osteoporosis screening and BMD testing in postmenopausal women < 65 years. In the BMI<25kg/m^2^ group, the over prevalence of osteoporosis and osteopenia was three times higher than in BMI in ≥25kg/m^2^ group. The study of Tang et al. indicated that the prevalence of osteoporosis was 69.9% in low weight people, 42.2% in normal weight subjects, 24.2% in overweight group and 14.6% in obese group [26]. Low weight was associated with higher prevalence of osteoporosis might because weight loss can cause a decrease in BMD [27]. For postmenopausal women with normal or low birth weight, osteoporosis and osteopenia should be prevented.

This study measured the trends of prevalence and treatment rate of osteoporosis in postmenopausal women based on the data from NHANES, and the samples are representative to some extent. The results might provide a reference for the management of osteoporosis in postmenopausal women. The sites for the detection of BMD were different in NHANES waves, and BMD of femur was commonly detected, while the BMD of lumbar spine were only collected from 2001–2002, and 2011–2018. BMD data at the femur neck have been proposed as the reference skeletal site for defining osteoporosis in epidemiological studies [28, 29]. BMD of femur was selected as the evaluation site for osteoporosis and osteopenia in our study, which might result in underestimation of the prevalence of osteoporosis and osteopenia. Similarly, physician diagnosed osteoporosis and treatment were obtained through questionnaires with possible recall bias. This was a cross-sectional study and a causal relationship of different characteristics with the risk of osteoporosis and osteopenia could not be identified.

## Conclusion

This study the trends of prevalence osteoporosis and osteopenia as well as the treatment rate of osteoporosis in postmenopausal women. The results showed a high prevalence of osteoporosis and osteopenia, but low treatment of osteoporosis in postmenopausal women. The prevalence of osteopenia presented an increased tread. The findings of our study implied the osteoporosis management might be insufficient and more efforts are needed to improve the diagnosis and treatment rates of osteoporosis in postmenopausal women.

## Supporting information

S1 Checklist STROBE statement—checklist of items that should be included in reports of observational studies. (DOCX)
